# Neonatal Occipital Exocranial and Endocranial Hamartoma at the University Teaching Hospital of Kinshasa: A Case Report

**DOI:** 10.7759/cureus.113400

**Published:** 2026-07-26

**Authors:** Christelle Ndjali, Glennie Ntsambi, Israël Maoneo, Ketani Teddy, Jean-Richard Kabongo, Ziko Punga, Michel Kisubi, Chérubin Tshiunza, Larrey Kasereka Kamabu, Antoine Beltchika

**Affiliations:** 1 Neurosurgery, University of Kinshasa, Kinshasa, COD; 2 Neurosurgery, Centre Hospitalier Initiative Plus, Kinshasa, COD; 3 Neurosurgery, Catholic University of Graben, Butembo, COD; 4 Neurosurgery, New Deal SARL Hospital/Clinique Internationale de Médecine Avancée Au Kivu (CIMAK), Goma, COD; 5 Neurosurgery, Makerere University, Kampala, UGA

**Keywords:** cerebral hamartoma, congenital disease, congenital malformation, neonatal surgery, neonate, occipital mass

## Abstract

Hamartoma is a rare benign malformation characterized by a disorganized but non-neoplastic proliferation of tissues normally found within the affected organ. Neonatal hamartoma is an uncommon condition of the central nervous system. We report the case of an 18-day-old female neonate brought by her family to the University Teaching Hospital of Kinshasa in the Democratic Republic of the Congo with a congenital occipital mass. On admission, the patient was alert, with stable vital signs and a preserved general condition. Physical examination revealed a 10 × 6 cm occipital mass, firm centrally and fluctuant at the periphery, with an irregular surface. The lesion was mobile relative to both superficial and deep planes and exhibited pulsatility. Cranial computed tomography revealed a heterogeneous extra-axial mass, initially suspected to be a teratoma. Complete surgical excision of the lesion was performed. Histopathological analysis confirmed the diagnosis of an exocranial and endocranial hamartoma. The postoperative course was complicated by a surgical site infection, which was successfully managed with broad-spectrum antibiotic therapy. Follow-up at three months was favorable. This case highlights an unusual occipital localization of hamartoma and underscores the importance of imaging and histopathological examination in the diagnosis and management of this congenital malformation.

## Introduction

A hamartoma is a rare benign malformation characterized by a disorganized but non-neoplastic proliferation of tissues normally found within the affected organ, although malignant transformation may occasionally occur [1]. It results from an embryological developmental anomaly and may involve various organs, including the lungs, kidneys, skin, and central nervous system [1-4]. Although often asymptomatic, a hamartoma may become clinically significant depending on its location, size, and functional impact.

Intracranial hamartomas are uncommon and are classically found in the hypothalamus, where they are linked to a wide range of neurological and endocrine manifestations [1-3,5]. In contrast to hypothalamic hamartomas (with an estimated incidence ranging from one in 50,000 to one in 1,000,000 individuals in the general population) [6], there is limited literature on isolated non-hypothalamic cerebral hamartomas (frontal, temporal, or occipital), particularly in neonates and children.

We report a case of occipital hamartoma and discuss the specific features related to its occipital location as well as its dual intra- and extracranial components.

## Case presentation

This was an 18-day-old female neonate, born at term via cesarean section indicated for cephalopelvic disproportion. Maternal history was notable for regular alcohol consumption during pregnancy, estimated at approximately 79 g per week.

The neonate was referred to the University Teaching Hospital of Kinshasa for the management of a congenital cystic-appearing mass located in the occipital region. On admission, the patient was alert, with stable vital signs and a preserved general condition.

Physical examination revealed a 10 × 6 cm occipital mass, firm centrally and fluctuant at the periphery, with an irregular surface. The lesion was mobile relative to both superficial and deep planes and exhibited pulsatility (Figure 1).

**Figure 1:**
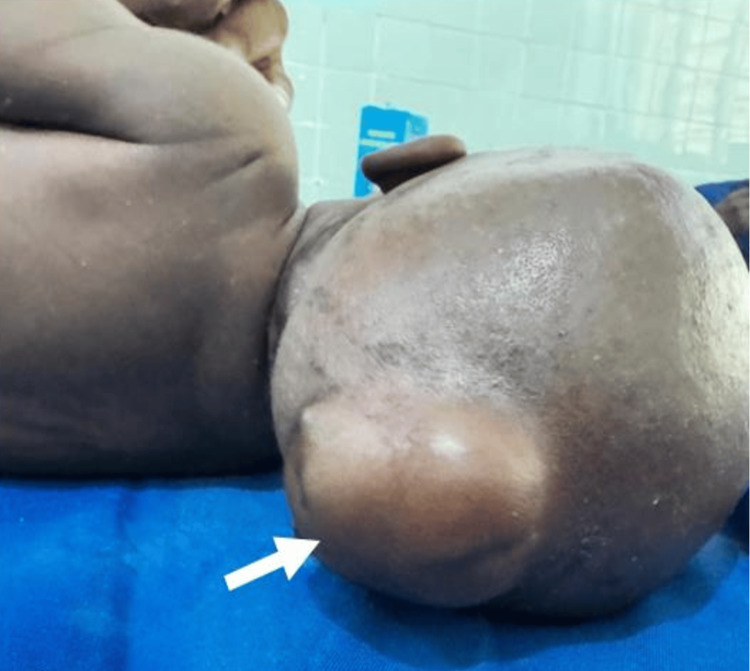
Occipital mass (white arrow), 10 × 6 cm in diameter, firm centrally and fluctuant at the periphery, with an irregular surface

The rest of the physical exam was unremarkable. The laboratory investigations (complete blood count, kidney and liver function tests) were within normal limits. Only the C-reactive protein was elevated at 26 mg/l. We considered a cystic lesion, a meningocele, or a meningoencephalocele. Due to financial constraints, the family was unable to afford magnetic resonance imaging (MRI). Therefore, contrast-enhanced cranial computed tomography (CT) was performed, revealing an endo- and exocranial mass measuring 40 mm at its largest axis, with heterogeneous density (a component made of bone, fluid, and fat, bounded by a thin, regular wall (Figure 2)), suggesting a congenital extra-axial mid-occipital abnormality, initially raising the possibility of a teratoma.

**Figure 2:**
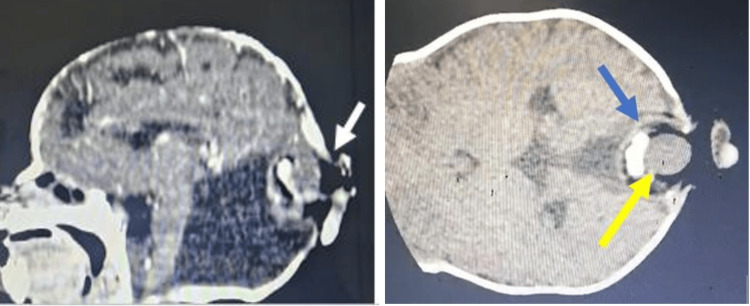
Sagittal slice of a brain CT scan showing the extra- and intracranial mass (white arrow). The axial slice shows the dura mater intact, unaffected by the mass (blue arrow), and the boundaries of the occipital bone, a mix of heterogeneous tissues (hypo- and hyperdense) (yellow arrow) CT: computed tomography

Surgical intervention was performed under general anesthesia, using a transverse incision centered over the lesion, extending approximately 2 cm on either side (Figure 3A). Dissection of the subcutaneous tissue allowed the visualization of the mass beneath the scalp, located in a supradural position (Figure 3B-3C).

**Figure 3:**
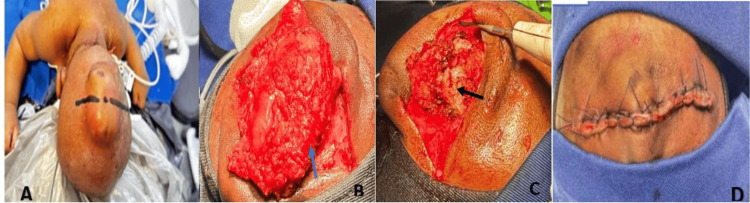
Intraoperative images: (A) incision line, (B) dissection of the subcutaneous tissue and exposure of the fleshy part of the mass (blue arrow), (C) the occipital bone is intact (black arrow), and (D) wound closure

Complete excision of the lesion was achieved. On gross examination, the mass was heterogeneous and composed of multiple tissue types, including solid (fleshy), osseous, cartilaginous, and fluid components (purulent content), with a total weight of 2 g (Figure 4).

**Figure 4:**
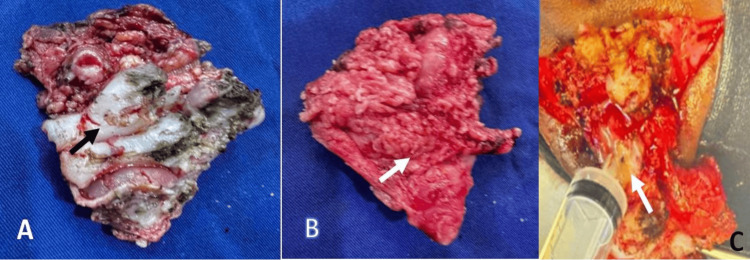
Resected heterogeneous mass including (A) osseous, cartilaginous (black arrow), (B) solid (fleshy) (white arrow), and (C) fluid components (purulent content) (white arrow), with a total weight of 2 g

The postoperative course was complicated by a surgical site infection, which was successfully managed with broad-spectrum antibiotic therapy and local wound care. At the three-month follow-up, the outcome was satisfactory, with favorable wound healing and preserved neurological status.

Histopathological examination revealed a poorly circumscribed benign mesenchymal proliferation composed of a heterogeneous mixture of mature cartilaginous tissue, fibrocollagenous stroma, and myxoid areas. No tumor necrosis, significant atypia, or features of aggressive invasion were identified. No malignant epithelial component was observed. The cartilaginous component consisted of well-differentiated chondrocytes embedded in regular lacunae, without cytonuclear atypia or mitotic activity. The surrounding stroma was fibrous to myxoid and sparsely cellular (Figure 5). We concluded with the histological diagnosis of an occipital mesenchymal hamartoma.

**Figure 5:**
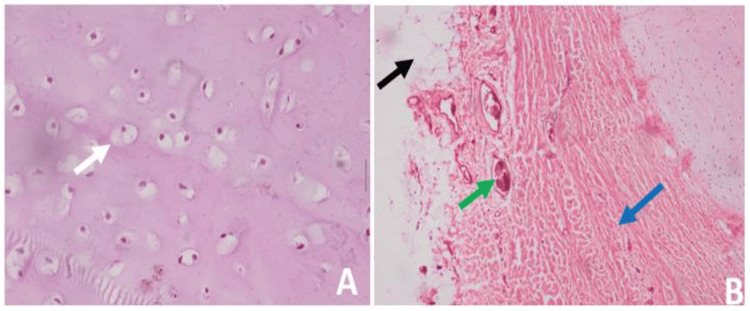
Histopathological images of the lesion showing (A) mature cartilaginous tissue with well-differentiated chondrocytes embedded within lacunae (white arrow) (H&E stain, ×25 magnification) and (B) myxoid tissue (black arrow) and fibrous stroma (blue arrow) containing a few congested blood vessels (green arrow) (H&E stain, ×25 magnification) H&E: hematoxylin and eosin

## Discussion

Within the central nervous system, hamartomas are most commonly located in the hypothalamus [5,6]. Non-hypothalamic cerebral hamartomas with both exo- and endocranial components, as observed in the present case, are rarely reported in the literature. In our case, the neonate presented with a midline occipital extra-axial lesion associated with a large congenital mass, representing an exceptional clinical presentation.

In neonates, the differential diagnosis of a congenital occipital mass includes encephaloceles, teratomas, dermoid cysts, meningoceles, posterior fossa anomalies, as well as other rare congenital tumors [7,8].

MRI remains the gold standard for the characterization of cerebral hamartomas, allowing the detailed assessment of anatomical relationships and tissue composition [2,6]. However, in our resource-limited setting, contrast-enhanced CT provided valuable initial diagnostic orientation and enabled appropriate surgical planning, underscoring the importance of accessible imaging modalities. Definitive diagnosis relies on histopathological examination, which demonstrates a disorganized arrangement of mature tissues without features of malignancy, as observed in our case [2].

Therapeutic management depends on clinical presentation, lesion size, and neurological impact [2]. In neonates, surgical intervention is generally indicated in cases of large masses, mass effect, risk of complications, or diagnostic uncertainty, as in our patient [2]. Complete surgical excision is associated with a favorable prognosis when the lesion is well circumscribed and surgically accessible.

Finally, the history of chronic maternal alcohol consumption during pregnancy, identified in this case, warrants particular attention. Although a direct causal relationship between prenatal alcohol exposure and hamartoma development has not been clearly established, alcohol is a well-known teratogenic factor implicated in numerous central nervous system developmental abnormalities [9,10]. This observation raises the question of a potential contributory role of prenatal alcohol exposure in congenital malformations, including hamartomas, and highlights an area for future research.

## Conclusions

This case report highlights an exceptional and little-documented location in the literature of a neonatal endo- and exocranial occipital mass. After the surgical removal of the tumor, histopathological analyses suggested a mesenchymal hamartoma. The immediate postoperative course was good. But the patient was lost to follow-up, making long-term monitoring impossible.
